# The Perspectives of Early Diagnosis of Schizophrenia Through the Detection of Epigenomics-Based Biomarkers in iPSC-Derived Neurons

**DOI:** 10.3389/fnmol.2021.756613

**Published:** 2021-11-12

**Authors:** Davin Lee, Jinsoo Seo, Hae chan Jeong, Hyosang Lee, Sung Bae Lee

**Affiliations:** Department of Brain and Cognitive Sciences, Daegu Gyeongbuk Institute of Science and Technology, Daegu, South Korea

**Keywords:** schizophrenia, iPSC, organoid, epigenetic alteration, transcriptional alteration

## Abstract

The lack of early diagnostic biomarkers for schizophrenia greatly limits treatment options that deliver therapeutic agents to affected cells at a timely manner. While previous schizophrenia biomarker research has identified various biological signals that are correlated with certain diseases, their reliability and practicality as an early diagnostic tool remains unclear. In this article, we discuss the use of atypical epigenetic and/or consequent transcriptional alterations (ETAs) as biomarkers of early-stage schizophrenia. Furthermore, we review the viability of discovering and applying these biomarkers through the use of cutting-edge technologies such as human induced pluripotent stem cell (iPSC)-derived neurons, brain models, and single-cell level analyses.

## Introduction

Neuropsychiatric diseases, such as autism, depression, and schizophrenia are neurological and psychiatric disorders that severely compromise diverse brain functions associated with normal activities. Despite decades of research, well-validated objective biological markers (measurable signals indicative of disease, infection, or injury) for neuropsychiatric diseases have remained stubbornly out of reach. This lack of objective biomarkers has relegated clinicians to diagnose neuropsychiatric diseases on the basis of phenomenological criteria (symptoms, signs, and course of illness), which, though useful in describing the disease in a rough and ready way, continues to produce heated controversies regarding the efficacy of properly diagnosing various, if not all, neuropsychiatric diseases (Bhati, 2013; Malaspina et al., 2013; Wakefield, 2016; Parker, 2019). In addition, the absence of biomarkers indicative of early-stage neuropsychiatric diseases undermines timely delivery of therapeutic agents to diseased neurons before neural impairment becomes so severe the damage cannot be reversed. Thus, perhaps unsurprisingly, temporally specific disease type-targeted treatment options for neuropsychiatric diseases remain largely unavailable.

The main cause for the lack of objective neuropsychiatric biomarkers is arguably in the absence of proper disease models (which can be used for studying/identifying early signals indicative of neuropsychiatric diseases) that accurately reflect the development and symptoms of early-stage neuropsychiatric diseases. Traditionally, modeling early-stage neuropsychiatric diseases has been exceptionally difficult as the model system must (1) reliably retain both genetic and environmental risk factors associated with neuropsychiatric diseases, (2) faithfully reproduce disease-specific early-stage neural aberrations, and (3) accommodate practical and ethical considerations. As such, these challenges have prevented the rigorous biological investigation of early-stage biomarkers for neuropsychiatric disorders.

Induced pluripotent stem cell (iPSC) and organoid technology are novel and emerging methods that largely bypass the difficulties laid out above. Hence, in this article, we highlight studies that model schizophrenia, a neuropsychiatric disorder characterized by episodes of psychosis, using patient-derived neural organoids. In addition, we propose utilizing single-cell level analyses in combination with organoid technology to discover disease-associated epigenetic and/or consequent transcriptional alterations (ETAs)-based biomarkers, a novel class of biomarkers previously proposed to be applicable in diagnosing neurodegenerative diseases (Lee et al., 2020). Finally, we discuss various obstacles in diagnosing asymptomatic patients using newly discovered ETA-based biomarkers and suggest solutions for these challenges.

## An Overview of Schizophrenia

Schizophrenia is a devastating psychiatric illness that affects approximately 1% of the population worldwide (Saha et al., 2008; Stilo and Murray, 2010). The disease consists of varying degrees of positive (hallucinations, delusions, disorganized speech and behavior, psychomotor agitation, and hypersensitivity to psychoactive drugs such as ketamine, phencyclidine, amphetamine, and cocaine), negative (social withdrawal, anhedonia, flattened affect, and alogia), and cognitive (deficits in attention, working memory, executive function, and reversal learning) symptoms (Perez and Lodge, 2014; Sigurdsson, 2016). The majority of these symptoms, aside from certain positive symptoms (Carbon and Correll, 2014; Leucht et al., 2020), currently have few or no effective pharmacological treatments.

Although the etiology of schizophrenia remains largely elusive, post-mortem studies of schizophrenic individuals have suggested defects in neurodevelopmental processes may be a cause. This is largely due to the discovery of macroscopic and histologic alterations in the brains of schizophrenic individuals. These alterations include: diminished brain volume, increased ventricle size, and abnormal cortical and hippocampal neural structures (Brown et al., 1986; Bogerts et al., 1993a,b), without clear pathological inclusion bodies, dystrophic neurites, or neurodegeneration (Wong and Van Tol, 2003). Hence, it remains possible that the detected structural abnormalities may not be directly considered a cause for schizophrenia, but merely a consequence of the disease.

Accumulating evidence suggests abnormal neural development caused by neurotransmitter dysfunction in multiple brain regions is likely to underpin schizophrenia (Lewis and Levitt, 2002; Fatemi and Folsom, 2009; Sigurdsson and Duvarci, 2015; Zamanpoor, 2020). The dopamine hypothesis, which remains the central dogma of schizophrenia for decades, posits that excessive subcortical dopamine signaling in the mesolimbic and nigrostriatal pathways is responsible for positive disease symptoms (Meltzer and Stahl, 1976; Weinberger, 1987; Davis et al., 1991; McCutcheon et al., 2019). However, the failure of dopamine blockage to relieve some patient symptoms suggests other neurotransmitters may play a role. Studies found that impaired glutamate signaling may also contribute to schizophrenia by causing functional deficits in inhibitory interneurons, resulting in disruption of the excitation-inhibition balance in the cortical and hippocampal regions (Benes and Berretta, 2001; Beasley et al., 2002; Hashimoto et al., 2003; Goto and Grace, 2005; Lodge and Grace, 2007; Lodge et al., 2009; Boley et al., 2014). This imbalance consequently leads to hyperactivity in the downstream dopamine system as well as abnormal neural circuit functions in other brain regions, which may culminate in the development of schizophrenia (Perez and Lodge, 2014; Sigurdsson, 2016). Lastly, some studies also implicate the serotonin pathway in schizophrenia development (Abdolmaleky et al., 2004; Alex and Pehek, 2007; Stepnicki et al., 2018). It is important to note, however, that the dopamine, glutamate, or serotonin hypotheses are not mutually exclusive and can be connected at the neural circuit level. Furthermore, studies suggesting neurotransmitter dysfunction contributes to schizophrenia also implicate that certain cell-types associated with the production and/or acquisition of neurotransmitters are central in the pathogenesis of schizophrenia.

While the underlying causes for abnormal neural development are currently unknown, genetic studies, including gene mapping, linkage analysis, genome-wide association studies, and next-generation sequencing, have uncovered schizophrenia susceptibility-associated single-nucleotide polymorphisms and schizophrenia-risk genes over the years (Zamanpoor, 2020). With a heritability rate of 79–81%, one may assume that schizophrenia can largely be explained through aberrations in genetic makeup and can be diagnosed through screening of key disease-associated genetic loci. Although genetically engineered animal models have reported evidence establishing the potential association between genetic risk factors and the disease (Winship et al., 2019), it is generally accepted that schizophrenia results from the interaction between increased susceptibility caused by genetic risk factors and environmental events that trigger the psychotic episodes (Ayhan et al., 2016). Thus, the diagnosis of schizophrenia through the screening of disease-associated genetic risk factors alone has proven to be extremely challenging. To diagnose schizophrenia properly, it is imperative that we find other ways to separate the healthy from the vulnerable.

## The Importance of Early Diagnosis of Schizophrenia

Because the treatment of schizophrenia and its symptoms are currently unavailable, scientists have alternatively attempted to alleviate the disorder by intervening the disease development in its early-stage, thereby ultimately preventing the accumulation of disease-associated neural impairments. This approach has garnered considerable attention as studies have consistently shown early identification and treatment of psychotic symptoms markedly improves clinical outcomes (Kulhara et al., 2008; Cheng and Schepp, 2016). For example, previous studies have shown a statistical correlation between prolonged Durations of Untreated Psychosis (DUPs) and poor clinical outcomes (McGlashan, 1999; Compton, 2004; Qin et al., 2014). Given the importance of early treatment for pre-symptomatic schizophrenic patients, a great deal of research has been conducted to find objective, well-validated, biomarkers for schizophrenia.

## The Necessity of Discovering New Biomarkers Specific for Early Stages of Schizophrenia

Recent efforts in search for schizophrenia biomarkers have yielded various potential signs that signal both disease presence and severity (Table 1). For example, neuroimaging-based studies have identified strong candidate biomarkers based on dopamine hyperactivity, hippocampal hyperactivity, immune dysregulation, *N*-methyl-D-aspartate receptor hypofunction, and cortical gray matter volume loss (Bogerts et al., 1990; Suddath et al., 1990; Shenton et al., 1992; Dierks et al., 1999; Hirayasu et al., 2000; Kasai et al., 2003; Koo et al., 2006; Ongur et al., 2006; Talati et al., 2014; Tregellas et al., 2014; Torres et al., 2016; Kraguljac et al., 2021). Additionally, research suggests some physiological complications observed in schizophrenic patients may also be used as potential biomarkers. For example, early auditory information processing (EAIP), has been proposed as a physiological biomarker of schizophrenia, as auditory discrimination-based testing is a highly quantitative and reliable measure of brain activity (Javitt et al., 1997; Perez et al., 2017). Finally, bio-fluids, such as blood (Lai et al., 2016) and cerebrospinal fluid (Huang et al., 2006), have been used as patient-derived sources for biomarkers to diagnose schizophrenia. Research indicates that the analysis of patient-derived blood anolytes (small molecules and proteins) distinguishes healthy controls from schizophrenic patients with up to 83% sensitivity and specificity (Schwarz et al., 2010). However, it is important to note that the biomarkers described above need significant improvements before being used generally. In the following sections, we will discuss what improvements must be made as well as potential ways in which these improvements can be achieved.

**TABLE 1:** Selected reports hinting potential schizophrenic biomarkers.

Sample origin	Technology	Biomarker class	Biomarker(s)	Sampling time frame	Subtype selectivity	References
Neuro-imaging	MRI	Brain volume loss	Gray matter volume loss in fronto-temporal cortex	Late-stage	None	Koo et al., 2006
			Temporal limbic structure volume loss	First episode SZ	None	Bogerts et al., 1990
			Decrease volume of hippocampus	First episode psychosis	None	Hirayasu et al., 2000
			Gray matter volume loss in amygdala	Late-stage	None	Shenton et al., 1992
			Gray matter volume loss in left Heschl gyrus and left planum temporale	First episode SZ	None	Kasai et al., 2003
			Gray matter volume loss in insula, inferior cortex, right superior and middle temporal cortices, left postcentral gyrus	Late-stage	None	Torres et al., 2016
		Ventricle enlargement	Enlarged lateral ventricles and third ventricle	Late-stage	None	Suddath et al., 1990
		Abnormal brain activity	Increase of the blood oxygen level-dependent signal in Heschl’s gyrus	Late-stage	Positive symptoms	Dierks et al., 1999
			Decrease of activation in the right parietal cortex and anterior cingulate cortex	Late-stage	Cognitive symptoms	Ongur et al., 2006
			Increased activity in right hippocampus	Late-stage	Negative, cognitive symptoms	Tregellas et al., 2014
			Increase of CBV in CA1 region of hippocampus	Late-stage	None	Talati et al., 2014
	PET imaging	Abnormal dopamine activity	Increase of dopamine synthesis capacity in the substantia nigra and striatum	Late-stage	Positive symptoms	Howes et al., 2013
			Hypoperfusion of dopamine in medial pre-frontal cortex to anterior cingulate cortex	Late-stage	Positive symptoms	Liddle et al., 1992
			Elevated F-dopa uptake in striatum	Prodromal	Positive symptoms	Howes et al., 2009
		Abnormal blood flow	Increase of rCBF in subcortical nuclei, hippocampus, parahippocampal, cingulate gyri	Late-stage	Positive symptoms	Silbersweig et al., 1995
	SPECT	NT dysregulation	Increase of dopamine transmission in striatum	Late-stage	Positive symptoms	Laruelle et al., 1999
			Increase of synaptic dopamine concentration in striatum	Ultra high risk for psychosis	Positive symptoms	Bloemen et al., 2013
			Reduction of NMDA receptors in the left hippocampus	Late-stage	Negative, cognitive symptoms	Pilowsky et al., 2006
			Increase of occupancy of D2 receptors in striatum	Late-stage	Positive symptoms	Abi-Dargham et al., 2000
	Proton MRS	NT dysregulation	Increase of glutamate level in hippocampus	Late-stage	Negative, cognitive symptoms	Kraguljac et al., 2013
Bio-fluids	ELISA	CSF	Increase of soluble IL-6 receptor level	Late-stage	Positive symptoms	Muller et al., 1997
			Increased IL-4 CSF level	Late-stage	None	Mittleman et al., 1997
		Blood	Increased IL-10 production	Late-stage	None	Cazzullo et al., 1998
			Increase plasma level of IL-2	Late-stage	Positive symptoms	Kim et al., 2000
			Decrease production of IFN-γ	Late-stage	None	Rothermundt et al., 1996
			IL-1, IL-6, TNF-α, vWf, OPG activity increase	Late-stage	None	Hope et al., 2013
			Increased protein expression of IL-6, TNF-α and mRNA level of IL-6, TNF-α, IL-1R1, TNFR1, TNFR2 in lymphocytes	Late-stage	None	Pandey et al., 2015
Proteome	Immunoblots	Abnormal protein expression	Increase protein expression of NR1 in anterior cingulate cortex	Late-stage	Negative, cognitive symptoms	Kristiansen et al., 2006
	*In situ* hybridization analysis	Transcriptional dysregulation	PSD95 transcript decrease in Brodmann area9	Late-stage	None	Ohnuma et al., 2000
Brain wave	EEG	Abnormal brain wave activity	Reduction of mismatch negativity amplitude in left the left hemisphere	Late-stage	Positive symptoms	Hirayasu et al., 1998
			Decreased Lempel-Ziv Complexity of EEG during mental activity	Late-stage	Cognitive symptoms	Li et al., 2008
			Abnormal Gamma-band Auditory steady-state responses latency	First-episode SZ	Cognitive symptoms	Tada et al., 2016
Patient sample	Electron microscopy	Reduced cellular organelle	The number of mitochondria reduction in anterior limbic cortex	Late-stage	None	Ong and Garey, 1993
Physiology	Hearing test	Abnormal physiological activity	Worsened tone matching performance	Late-stage	None	Javitt et al., 1997
Post-mortem sample	Post-mortem studies	NT dysregulation	Increase of postsynaptic dopamine receptor sensitivity in nucleus accumbens, putamen, caudate nucleus	Post-mortem	Positive symptoms	Owen et al., 1978
			Increase of presynaptic D2 auto-receptor in dorsolateral prefrontal cortex	Post-mortem	Positive symptoms	Kaalund et al., 2014
			Elevated dopamine D4 receptors density in striatum	Post-mortem	Positive symptoms	Seeman et al., 1993
		Abnormal mRNA expression	Increase mRNA expression of NR1, NR2A in dorsolateral prefrontal cortex and occipital cortex	Post-mortem	None	Dracheva et al., 2001
		Dendritic spine abnormalities	Reduction of dendritic spine density in frontal cortex and temporal cortex	Post-mortem	None	Garey et al., 1998
			Decrease of dendritic spine size in striatum	Post-mortem	None	Roberts et al., 1996
		Reduced cellular organelle	Reduction in both number and volume of mitochondria of oligodendroglia in the caudate nucleus and prefrontal cortex	Post-mortem	None	Uranova et al., 2001
			Decrease of density of mitochondria in the neuropil	Post-mortem	None	Kung and Roberts, 1999
		Reduced cellular volume	Reduction in somal volume of pyramidal cells in auditory association cortex	Post-mortem	None	Sweet et al., 2003

*SZ, schizophrenia; MRI, magnetic resonance imaging; EEG, electroencephalography; MRS, magnetic resonance spectroscopy; ELISA, enzyme-linked immunosorbent assay; SPECT, single-photon emission computed tomography; CBV, cerebral blood volume; rCBF, regional cerebral blood flow; NT, neurotransmitter; CSF, cerebrospinal fluid.*

The primary concern with the biomarkers described previously is their questionable utility as a schizophrenia biomarker in the early stages of disease development. While the latest renditions of schizophrenia biomarkers are becoming increasingly practical and accurate, most, if not all, current biomarkers are inapplicable to pre-symptomatic patients. Schizophrenia, as well as many other neurological diseases, is easily identifiable in structured interviews in its post-onset stages. Therefore, biomarkers that are used in post-onset patient-derived samples, run the risk of being redundant by nature, unless the biomarker can provide additional information on top of the rough and ready interview-based diagnosis. For instance, biomarkers high in specificity can distinguish schizophrenia by subtype and help decide the administration of patient-specific treatments. Instead, to fully exploit the utility of biomarkers, they should be used to detect disease-specifying, ideally disease subtype-specifying, signals before the phenotypic onset occurs to administer disease-specific therapeutics as early as possible. For this, it is not only essential that the selected biomarkers themselves be observable in early-stage schizophrenia, but also necessary that such biomarkers be found in specific cell-types. We have named these biomarker containing cells first-responding disease-associated cell types (FDCs).

To achieve this, we suggest the use of disease-associated ETAs be used as early-stage schizophrenia biomarkers, as they have high potential to be exploited as an early signal for schizophrenia. Additionally, we propose that (1) these biomarkers and FDCs should be discovered in patient-derived neural organoids (such as synthesized miniature brains) and (2) found biomarkers should be applied in discovered FDCs generated through patient-derived iPSCs. By using these cutting-edge methods, we believe reliably identifying pre-symptomatic schizophrenic patients is possible. We further delineate our proposal in the following sections.

## Epigenetic and/or Consequent Transcriptional Alterations as Schizophrenia Biomarkers

Before the first onset of a frank schizophrenic episode, patients experience clinical features such as mild physical anomalies, poor motor coordination, mild cognitive impairments, social deficits, anxiety, sadness, lability, irritability, sleep disturbances, cognitive impairment in attention, concentration, mild illusions, suspiciousness, magical thinking, substance use, social withdrawal, and preoccupations (McGlashan, 1996; McGlashan and Johannessen, 1996; Lieberman et al., 2001). Since these premorbid and prodromal pathological symptoms are generally mild in nature, it is no surprise that these symptoms cannot be considered as biomarkers for diagnostics (Erlenmeyer-Kimling and Cornblatt, 1987). This becomes particularly clear when considering these phenomena largely overlap with range of mental and behavioral experiences of young adults who do not subsequently develop schizophrenia.

Research, however, indicates that pre-symptomatic patients undergo a series of neurological perturbations that render the brain at “high-risk.” The pathophysiological processes during the premorbid and prodromal stages are largely categorized as neurodevelopmental (inductive, patterning, and synaptogenetic anomalies) and neuroplastic (post-pubertal hormonal effects, myelination, and synaptic regression) dysfunctions. Both pathological dysfunctions and their phenotypic manifestations are highly diverse, suggesting that the biological mechanism underpinning such broad aberrations may be associated with system-wide atypical cellular changes in certain specific disease-associated cell-types, such as FDCs. Here, ETAs are highlighted as alterations in ETAs are likely responsible for these system-wide changes.

It must be pointed out that research on the early stages of schizophrenia is mostly incomplete. Perhaps the most obvious reason underlying this shortcoming is in the lack of disease models that retain genetic and environmental risk factors associated with schizophrenia while validly reproducing early-stage biological insults. Animal models of schizophrenia, while can be helpful in investigating some contributions of schizophrenia-associated genetic aberrations and/or environmental factors, are largely inadequate in recapitulating the complex, neuron-specific, interactions between genetic, epigenetic, and transcriptional factors observed in schizophrenic patients.

## Patient-Derived Induced Pluripotent Stem Cell for Modeling Schizophrenia

These complications have led researchers to model schizophrenia using patient-derived iPSC cellular models. Schizophrenia iPSC lines were first established from Disrupted in schizophrenia 1 (DISC1) mutation-carrying chronic paranoid schizophrenia patient in 2011 (Chiang et al., 2011). Since then, iPSC and its derivative technologies, such as patient-derived organoids, have steadily enabled a wide range of studies investigating schizophrenia. We have summarized key studies that modeled schizophrenia using iPSC or its derivative technologies in Table 2.

**TABLE 2:** Selected reports utilizing iPSC models and organoid technologies for the characterization of schizophrenia.

Sample origin		*In vitro* model	Observation(s)	Implication(s)	Sampling time frame	Subtype selectivity	References
fSZ/sSZ	Fibroblasts	iPSC, differentiated to neural progenitor cells	Abnormal gene and protein levels related to cytoskeletal remodeling and increased oxidative stress	SZ hiPSC NPCs may serve as a proxy for the developmental pathways potentially contributing to SZ pathogenesis	Late-stage	None	Brennand et al., 2015
	Fibroblasts	iPSC, differentiated to CA3 neurons and DG neurons	Reduction of activity in DG-CA3 co-culture and deficits in spontaneous and evoked activity in CA3 neurons	iPSC-derived CA3 neurons and DG neurons may recapitulate SZ hippocampal activity	Late-stage	None	Sarkar et al., 2018
	Fibroblasts	iPSC, differentiated to neural progenitor cells, glutamatergic neurons	Reduced number of neurite and protein level of PSD95	Report decreased neuronal connectivity, synaptic protein level and glutamate receptor expression in SZ iPSC neurons	Late-stage	None	Brennand et al., 2011
	Fibroblasts	iPSC, differentiated to dentate gyrus granule neurons	Reduced hippocampal neurogenesis and attenuated spontaneous neurotransmitter release	iPSC-derived DG granule neurons recapitulate SZ hippocampal neurogenesis features including lowered level of NEUROD1, PROX1 and TBR1	Late-stage	None	Yu et al., 2014
	Fibroblasts	iPSC, differentiated to forebrain mixed neurons	Increase of gene expression of WNT signaling genes and β-catenin protein levels	Abnormal expression of WNT signaling proteins may underlie SZ	Late-stage	None	Topol et al., 2015
	Fibroblasts	iPSC-derived cerebral organoids	Altered distribution of cortical neurons in correlation with abnormal expression of nuclear FGFR1 in neuronal committed cells	Altered FGFR1 signaling is linked to cortical malformation in SZ patient-derived cerebral organoid models	Late-stage	None	Stachowiak et al., 2017
	Fibroblasts	iPSC-derived cerebral organoids	Increased production of malformative TNF and increased developmental vulnerability to TNF	Development of brain pathology in SZ is exacerbated by abnormal TNF expression	Late-stage	None	Benson et al., 2020
fSZ	Fibroblasts	iPSC, differentiated to neural progenitor cells	Reduced protein level of CYFIP1 and WAVE	15q11.2 microdeletion affects neural developmental processes by altering CYFIP1 signaling and WAVE complex signaling	Childhood-onset SZ	15q11.2 deletion	Yoon et al., 2014
	Fibroblasts	iPSC, differentiated to neurons, neural progenitor cells and oligodendrocyte precursor cells	Decreased expression of exon14-15 of CNTNAP2 and reduced neural migration	Present that both exon-and allele-specific expression of CNTNAP2 may be critical for the predisposition of SZ	Late-stage	Large heterozygous CNTNAP2 deletion	Lee et al., 2015
	Hair follicle keratinocytes	iPSC, differentiated to dopaminergic neurons and glutamatergic neurons	Morphological abnormalities of dopaminergic neurons and mitochondrial dysfunction in keratinocytes	Unravel impaired ability to differentiate into dopaminergic neurons and mitochondrial dysfunction including abnormal mitochondrial membrane potential, trafficking and turnover in SZ	Late-stage	Paranoid SZ	Robicsek et al., 2013
	Fibroblasts	iPSC, differentiated to neural progenitor cells	Downregulation of miR-9 in correlation with reduced migration in neural progenitor cells	miR-9 as a risk factor for SZ	Late-stage and childhood-onset SZ	None	Topol et al., 2016
sSZ	Fibroblasts	iPSC, differentiated to glutamatergic and GABAergic neurons	Reduced expression of almost all genes in the 22q11.2 region	Identified potential druggable targets in neuropsychiatric diseases associated with 22q11.2 del	Late-stage	22q11.2 deletion	Lin et al., 2016
	Fibroblasts	iPSC, differentiated to neural progenitor cells and neurospheres	Reduced expression level of miRNAs belonging to miR-17/92 cluster and miR-106a/b and upregulated p38α	Potential novel p38α-targeting therapeutics for SZ	Late-stage	22q11.2 deletion	Toyoshima et al., 2016
	Fibroblasts	iPSC, differentiated to mixed neurons	Dysregulated expression of miRNAs	Suggest that iPSCs derived from patient with 22q11.2 del SZ may be utilized to study the disease	Late-stage and childhood-onset SZ	22q11.2 deletion	Zhao et al., 2015
	Fibroblasts	iPSC, differentiation to forebrain mixed neurons and MAP2AB + neurons	Reduced wild-type DISC1 protein level and synaptic vesicle release	Mutant DISC1 depletes wild-type DISC1 protein and causes synaptic abnormalities including defects in glutamatergic synaptic transmission and synapse formation	Late-stage	DISC1 4-bp deletion	Wen et al., 2014
	Fibroblasts	iPSC, differentiated to neural precursor cells	Increase of extramitochondrial oxygen consumption and ROS level	Abnormal metabolic changes during neurogenesis is associated with SZ	Late-stage	Clozapine resistant patient	Paulsen Bda et al., 2012

*fSZ, familial schizophrenia; sSZ, sporadic schizophrenia; DG, dentate gyrus; TNF, tumor necrosis factor; ROS, reactive oxygen species.*

No matter how advanced iPSC modeling of schizophrenia becomes, the utility of iPSC or its derivative technologies as a method to sample ETA-based biomarker sources relies on whether patient-derived samples have measurable differences in epigenetic, or other transcriptional profiles, as compared to healthy controls. Increasingly, more and more studies continue to provide strong evidence for this notion. For example, an early study by Brennand et al. (2011) showed that differentiated iPSC neurons from a schizophrenic patient had impaired neuronal connectivity, decreased neurite number, expression of synaptic proteins such as PSD-95, and, importantly, altered transcript expression. Similarly, multiple studies have shown reduced levels of neuronal differentiation-associated genes in neural progenitor cells from schizophrenic patient-derived iPSCs (Yu et al., 2014), suggesting a link between altered microRNA profiling (Zhao et al., 2015; Topol et al., 2016; Akkouh et al., 2021), chromatin openness in which disease variants affecting neurodevelopment reside (Forrest et al., 2017; Zhang et al., 2020), and protein expression patterns (Tiihonen et al., 2019). These results demonstrate (1) iPSC-derived neurons, but not other cell-types, from schizophrenia patients faithfully recapitulate many pathological disease features and (2) iPSC-derived neurons may be used to detect uniquely altered transcriptional, epigenetic, and proteome signals that could be utilized as potential biomarkers.

The fact that ETAs are observed in iPSC lines differentiated into neurons, but not other non-disease-associated cell- types strongly suggests that schizophrenia primarily targets neural cells, which, as previously mentioned above, is already widely accepted (Iritani, 2013). This becomes particularly important in the search for cell-types that exhibits early manifestation of measurable ETA-based signals because it suggests that among various cell-types intercommunicating within the brain, neurons are particularly sensitive to schizophrenia-associated perturbations. Here, single-cell level analyses are highlighted, as these technologies, combined with patient-derived organoids, could allow investigations into various specific cell-subtypes in a wide range of brain cells early in its disease development. In fact, single-cell level analyses have already attracted attention as methods that allow improved characterization of schizophrenia (Skene et al., 2018; Sawada et al., 2020). Bulk-level tissue sampling provides limited resolution for identifying cell-type-specific abnormalities. However, with single-cell analysis, cells are characterized by their RNA and/or transcriptome and grouped by their cell state and type. Exemplifying the capability of single-cell RNA sequencing analysis, Sawada et al. (2020), showed developmental excitation-inhibition imbalance in neural organoids derived from monozygotic twins discordant for psychosis. The authors further demonstrated that GABAergic neurons, but not other cell-types, showed alterations in their RNA profiles. Interestingly, these results suggest iPSC-derived GABAergic neurons may be FDCs that could be used to identify ETA-based biomarkers, such as those observed by Sawada et al. (2020). Collectively, we suggest that the discovery of both ETA-based biomarkers and FDCs is possible through the combined use of organoid technology and single cell-level analyses.

## Potential Limitations in the Practicality of Diagnosing Asymptomatic Schizophrenic Patients Using Discovered Epigenetic and/or Consequent Transcriptional Alteration-Based Biomarkers

For our strategy to be used as a method to diagnose schizophrenia to the general public, it is important to consider the cost of such approach. Unfortunately, iPSC generation and single-cell sequencing are among the most difficult and expensive techniques in modern biomedicine. To put it in numbers, iPSC reprogramming of 20,000 cells costs an estimated $3,000 ∼ $9,000 per sample ($0.1.5 ∼ $0.45 per cell). On top of that, if the generation of iPSC-derived neurons produces not only pure FDCs but also heterogeneous cell-types, each sample will cost an additional estimated $2,000 ∼ $8,000 for single-cell analysis. Predicting the cost of our strategy is further complicated by the fact that the expenditure of iPSC generation and single-cell sequencing is determined by various relevant factors. For example, the cost of iPSC generation is determined by the source of peripheral cell (fibroblast, blood cell), reprogramming methods (viral, non-viral) as well as the target differentiation cell-type (cerebral organoid, mid-brain, dopaminergic neuron, GABAergic neuron). The cost of single-cell sequencing is determined by protocols involving the preparation of cells (methods such as single-nucleus RNA sequencing, drop-sequencing), the number of cells analyzed per sample, as well as the number of sequencing reads per cells. If the price of our method is too high, the clinical application loses practical merit regardless of the prospects of diagnosing schizophrenia in its early phase.

**FIGURE 1:**
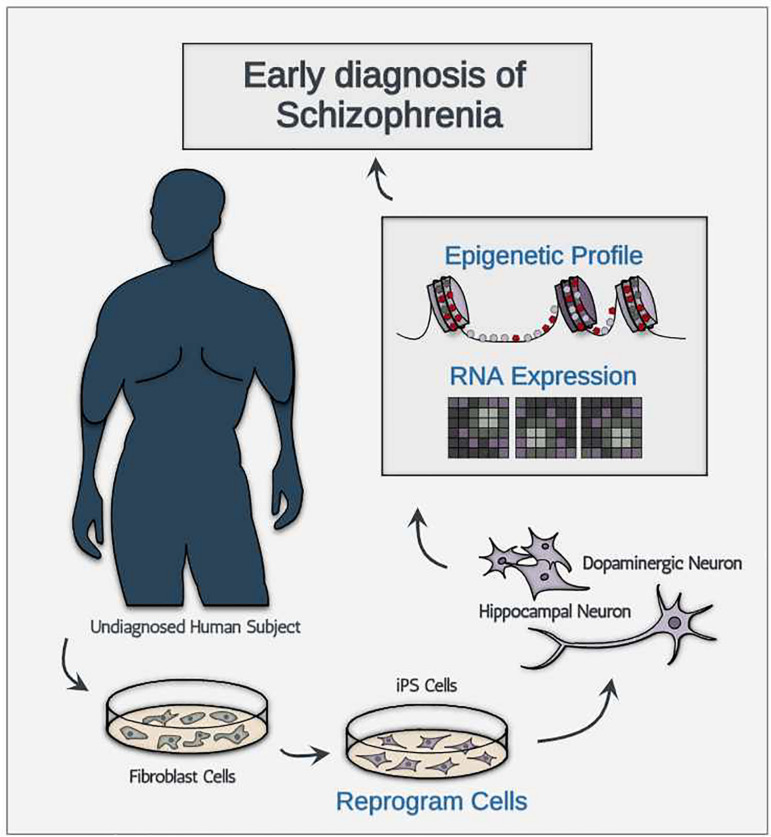
A schematic illustration showing the conceptual diagnostic workflow consisting of iPSC-derived neurons for early diagnosis of schizophrenia.

Despite these obstacles, we are still optimistic in predicting that the technologies will decrease in price for the following three reasons. First, much like many other technological advances, the price for newly developed technologies will likely decrease after the quality of product reaches a certain threshold. Between now and when iPSC generation technology and single-cell sequencing were first introduced (2006 and 2009, respectively; Takahashi and Yamanaka, 2006; Tang et al., 2009), the technological advancement has been focused in increasing both quality and purity of product, instead of decreasing the cost of the technologies. Therefore, to project the cost of single-cell sequencing and iPSC generation out into the future with past data points in cost may be mis-leading. Second, compiled meta-analysis and data sharing technologies may help bypass additional sampling required for the discovery of new ETA-based biomarkers. Third, assuming that technological advancement reaches to a point where reliable generation of pure FDCs are produced at a high quantity, diagnosing asymptomatic schizophrenic patients would only require targeted generation of FDCs using cell type-specific iPSC without the use of expensive single-cell analysis technologies (Figure 1). To sum, we believe that the cost concern surrounding iPSC generation and single-cell level analysis is quite manageable given the rapid technological advancement we have seen in the past decade.

## Discussion

Despite the clear advantages of biomarkers, psychiatrists and clinicians have yet to adopt any of the previously devised neuroimaging-, physiological-, or blood sample-based biomarkers for diagnosing schizophrenia. As noted by the developers of a blood sample-based biomarker for schizophrenia (VeriPsych), this reluctance may be due to the fact that “most psychiatrists believe that they are very good at diagnosing schizophrenia patients using a basic clinical interview” (Bahn et al., 2011). Unfortunately, this shows that (1) research has failed to generate a practically applicable early-stage biomarker that is more reliable in diagnosing schizophrenia than structured interviews and (2) clinicians are largely not benefiting from diagnosing schizophrenia based on raw biological data (which can specify schizophrenia by subtype and help decide the administration of patient-specific treatments). It is for these reasons we developed our strategy to practically meet the demands of practitioners who treat schizophrenia. Although our proposed strategy may help early diagnosis and, thus, intervention of schizophrenia, there are potential concerns surrounding our strategy. Below, we describe and address these concerns.

Given that our proposed strategy requires ETA-based biomarkers originating from disease-associated genetic aberrations, our model could be considered only applicable to inherited forms of schizophrenia. From this consideration, two concerns emerge: (1) the redundancy of ETA-based biomarkers and (2) the limited generalizability of our strategy. Concerns about the redundancy of ETA-based biomarkers stem from a pragmatic point of view where reliable early schizophrenia diagnosis is likely best achieved through a concerted effort combining multiple biomarkers including: genomics, physiology, and ETAs. However, if ETA profile alterations are observed only in inherited schizophrenia cases and abnormal ETA profiles develop from genetic differences between healthy and schizophrenic patients, identifying high-risk schizophrenic patients could be achieved by simply screening genetic loci associated with schizophrenia without using ETA-based biomarkers. While the concern surrounding the redundancy of our proposal is, to an extent, legitimate, careful examination of ETA-based biomarkers may provide significant advantages over traditional genomics-based schizophrenia diagnosis.

For instance, biomarkers that exploit altered disease-specific ETA profiles may help identify high-risk schizophrenic patients with hidden or otherwise unknown genetic risk factors. While genome-wide association studies have identified more than 100 schizophrenia susceptibility genes and predicted more than 1,000 (Shaw et al., 1998; Schizophrenia Working Group of the Psychiatric Genomics Consortium, 2014; Li et al., 2017; Pardinas et al., 2018), the rate to which familial genetic aberrations are linked to phenotypic psychosis is strikingly low despite the near 80% inheritance rate when either parent is diagnosed with schizophrenia (Cardno and Gottesman, 2000). This implies that not only is screening for genetic loci associated with schizophrenia not practical but that some cases of inherited schizophrenia are caused by currently unknown genetic changes. ETA-based biomarkers may be effective in identifying high-risk schizophrenic patients that were assigned low-risk using genetic screening alone, assuming these unknown genetic changes lead to disease-associated ETAs.

In fact, the advantages of ETA-based biomarkers over traditional genomics-based biomarker are also closely connected to the concerns about the possible limited generalizability of our strategy. Schizophrenia affects approximately 1% of the population worldwide. About 40% of the disease is thought to be familial cases, with the other 60% occurring sporadically in individuals with no family history of the disease. Interestingly, research indicates that approximately 10% of these sporadic cases are linked to *de novo* germline mutations (Xu et al., 2008). This suggests that, if these various *de novo* germline mutations can produce stereotypical, easily measurable, alterations in ETA profiles, ETA-based biomarkers could be used to identify sporadic schizophrenia cases. It is worth pointing out, however, that since it is currently unknown how much, and how distinct, disease-specific genetic abnormalities translate directly into alterations in ETA profiles, further studies investigating the unique ETA profiles of schizophrenic patients are needed.

In this review, we have presented our perspectives on the discovery and the practical application of ETA-based biomarkers for early diagnosis of schizophrenia based on recent advances in patient-derived iPSC and organoids. Although potential issues such as initial high cost may prevent widespread use of ETA-based biomarkers, we are firmly optimistic that our proposed strategy will be generally useful, especially considering the rapid pace at which these technologies continue to advance.

## Author Contributions

DL, JS, HL, and SL conceptualized the theme of the review and wrote the manuscript together. HJ contributed to Tables 1, 2. All authors contributed to the article and approved the submitted version.

## Conflict of Interest

The authors declare that the research was conducted in the absence of any commercial or financial relationships that could be construed as a potential conflict of interest.

## Publisher’s Note

All claims expressed in this article are solely those of the authors and do not necessarily represent those of their affiliated organizations, or those of the publisher, the editors and the reviewers. Any product that may be evaluated in this article, or claim that may be made by its manufacturer, is not guaranteed or endorsed by the publisher.
